# Pulmonary Nocardiosis in an Immunocompetent Patient: A Case Report

**DOI:** 10.1002/ccr3.70505

**Published:** 2025-06-05

**Authors:** Reza Ibrahimi, Farid Poursadegh, Mahnaz Mozdourian, Fariba Rezaeetalab, Zohre Emamdadi, Sahar Arab Yousefabadi, Sara Samadi

**Affiliations:** ^1^ Faculty of Medicine Mashhad University of Medical Sciences Mashhad Iran; ^2^ Lung Diseases Research Center Mashhad University of Medical Sciences Mashhad Iran; ^3^ Department of Pathology Emam Reza Hospital, Mashhad University of Medical Sciences Mashhad Iran; ^4^ Student Research Committee Mashhad University of Medical Sciences Mashhad Iran; ^5^ Department of Internal Medicine, Faculty of Medicine Mashhad University of Medical Sciences Mashhad Iran

**Keywords:** *Mycobacterium tuberculosis*, nocardia, pneumonia, therapy

## Abstract

The patient, a 56‐year‐old female with a history of childhood bronchiectasis and controlled hypertension, presented with fever, cough, and hemoptysis. Despite initial treatment for exacerbation of bronchiectasis, her condition worsened, leading to sepsis. Blood tests revealed leukocytosis and elevated CRP. Lung HRCT showed necrotic consolidation and nodules in the left and right lung, suggestive of necrotizing pneumonia. Bronchoscopy revealed *Nocardia* species, leading to intravenous co‐trimoxazole treatment. After a week in ICU, the patient improved and was discharged with oral co‐trimoxazole. The patient remained free of relapse during a 6‐month follow‐up period, with no CNS or cutaneous involvement detected.

**Summary**

- Nocardiosis is caused by a weakly acid‐fast bacillus found in fresh and salt water, decaying vegetables, soil and animal waste.
- Nocardiosis, primarily presenting as pneumonia, can manifest with CNS or cutaneous involvement, particularly in immunocompromised individuals but it can be present in immunocompetent cases.
- In endemic *Mycobacterium tuberculosis* ( *M. tuberculosis* ) regions like Iran, *Nocardia* can mimic *M. tuberculosis* symptoms, posing diagnostic challenges due to their similar acid‐fast staining properties.
- The antibiotic therapy, based on local resistance patterns, yielded an adequate response.
- Bronchoscopy and specimen assessment are valuable in diagnosing non‐responsive pneumonia, regardless of immune status.

## 1 Introduction

Nocardiosis, caused by *Nocardia*, a weakly acid‐fast [1], gram‐positive filamentous bacillus bacterium with over 50 known species, is often regarded as an opportunistic infection, even though approximately one‐third of nocardiosis patients are immunocompetent [2]. *Nocardia* spp. can be found in fresh and salt water, decaying vegetables, soil, and animal excreta. Noteworthy risk factors predisposing individuals to nocardiosis infections encompass conditions such as bronchiectasis, HIV infection, malignancies, immunosuppressive therapy, and diabetes mellitus [3, 4]. The comprehensive understanding of the true epidemiological aspects of *Nocardia* remains unclear in the existing literature. Reports on the incidence of *Nocardia* infections indicate its presence in multiple organs, affecting the pulmonary system, cutaneous tissues, and the central nervous system (CNS). This pathogenic microorganism is prevalent in various regions globally, including but not limited to Italy, India, Pakistan, and Iran [5, 6].

Pulmonary nocardiosis often occurs more commonly in immunocompromised individuals, unlike cutaneous nocardiosis, which mainly affects immunocompetent patients. It is important to note that pulmonary nocardiosis is the most common form of infection [7, 8].

The diagnosis of nocardiosis is achieved through clinical, radiological, microbiological, and laboratory examinations of isolated specimens. The majority of diagnoses rely on microscopic and phenotypic characteristics. Classic indicators of nocardiosis involve abscess formation and a lack of response to conventional antibiotic treatments, including community acquired pneumonia (CAP). Additionally, recurrent infections that persist despite antimicrobial therapy are commonly noted. Furthermore, the varied clinical presentations associated with nocardiosis have led to its characterization as “the great masquerader” [9].

## 2 Case History

The subject under consideration, a 56‐year‐old female, presented with a 2‐week history of fever, cough, and non‐massive hemoptysis at the time of the medical visit. Notably, she had a medical history indicative of childhood bronchiectasis of post‐infectious origin, which was effectively managed through as‐needed bronchodilator therapy. Concurrently, the patient had a controlled history of hypertension managed with angiotensin‐converting enzyme (ACE) inhibitors. Importantly, she was a non‐smoker and had no record of immune system deficiencies. During the initial outpatient visit, the physical examination unveiled a blood pressure (BP) of 110/80 mmHg, a pulse rate (PR) of 90 beats per minute, a respiratory rate (RR) of 14 breaths per minute, and an oral temperature (T) of 38.5°C. The room air oxygen saturation (SpO2) level was recorded at 96% in ambient air. The patient's body mass index (BMI) measured 23 kg/cm^2^.

## 3 Methods

The patient was treated with levofloxacin under the impression of a bronchiectasis exacerbation. However, after a week of appropriate antibiotic therapy, the patient experienced aggravated dyspnea and a worsening of previous symptoms. Consequently, she sought emergency medical attention and was admitted due to an acutely deteriorating clinical condition. Upon admission, the patient presented as ill and septic, with the following vital signs recorded: BP of 110/80 mmHg, PR of 110 beats per minute, RR of 30 breaths per minute, T of 39°C, and SpO2 of 86%.

## 4 Conclusions and Results

Following the initial laboratory tests, blood culture results were negative, and polymerase chain reaction (PCR) assays for influenza and COVID‐19 yielded negative results. The complete blood count (CBC) revealed leukocytosis at 21,000 white blood cells (WBC) per microliter, with a significant increase t in neutrophils (85.4%). Furthermore, the C‐reactive protein (CRP) level was notably elevated (132 mg/L).

**FIGURE 1:**
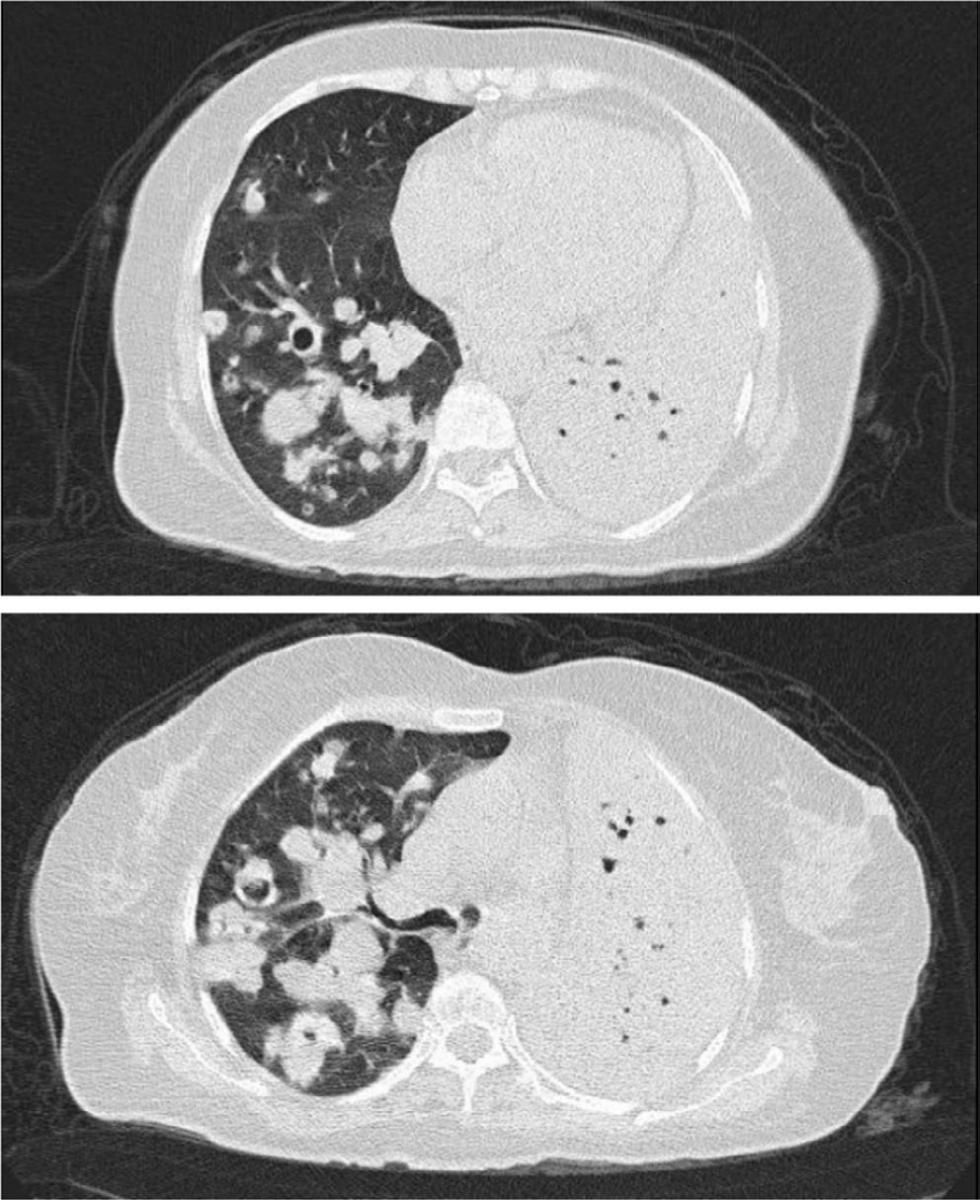
Diffuse consolidation in the left lung and scattered excavated nodules in the right lung.

Considering her condition, a high‐resolution computed tomography (HRCT) scan of the lungs was performed. The results indicated necrotic consolidation and opacities in the left lung, along with scattered nodules showing an excavated appearance in the right lung (Figure 1).

**FIGURE 2:**
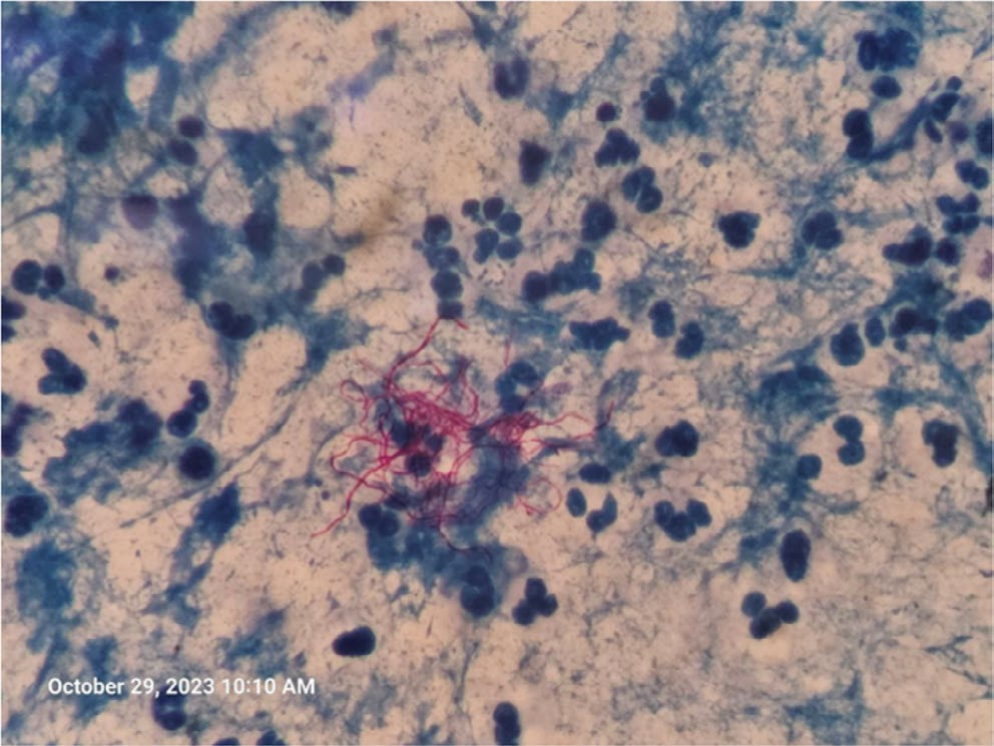
Modified Ziehl‐Neelsen staining showing acid‐fast branched bacilli in favor of nocardiosis.

In light of these diagnostic findings, the patient was initiated on a broad‐spectrum antibiotic regimen consisting of meropenem and vancomycin to address the suspected necrotizing pneumonia. However, given the persistent respiratory distress and a decrease in oxygen saturation, the patient was transferred to the intensive care unit (ICU). After 48 h of continuous antibiotic treatment, the patient still presented febrile symptoms, leading to a bronchoscopy to delve deeper into the case of “non‐responding pneumonia.” The bronchoscopy results revealed negative results for *M. tuberculosis* PCR, common viral infections, and galactomannan. Notably, Gram stain revealed weakly acid‐fast positive bacilli, and subsequent culture analysis confirmed the presence of *Nocardia* species (Figure 2).

Given the patient's clinical condition and the recent diagnosis, intravenous co‐trimoxazole was administered. A brain magnetic resonance imaging (MRI) showed no signs of CNS nocardiosis.

After a week, the patient showed signs of improvement, including the resolution of fever, which resulted in her discharge from the ICU. She also resumed oral intake, facilitating a switch to oral co‐trimoxazole.

Upon discharge, an investigation for immunodeficiency was conducted: she tested negative for HIV, and serum protein electrophoresis and immune‐electrophoresis were within normal limits.

**FIGURE 3:**
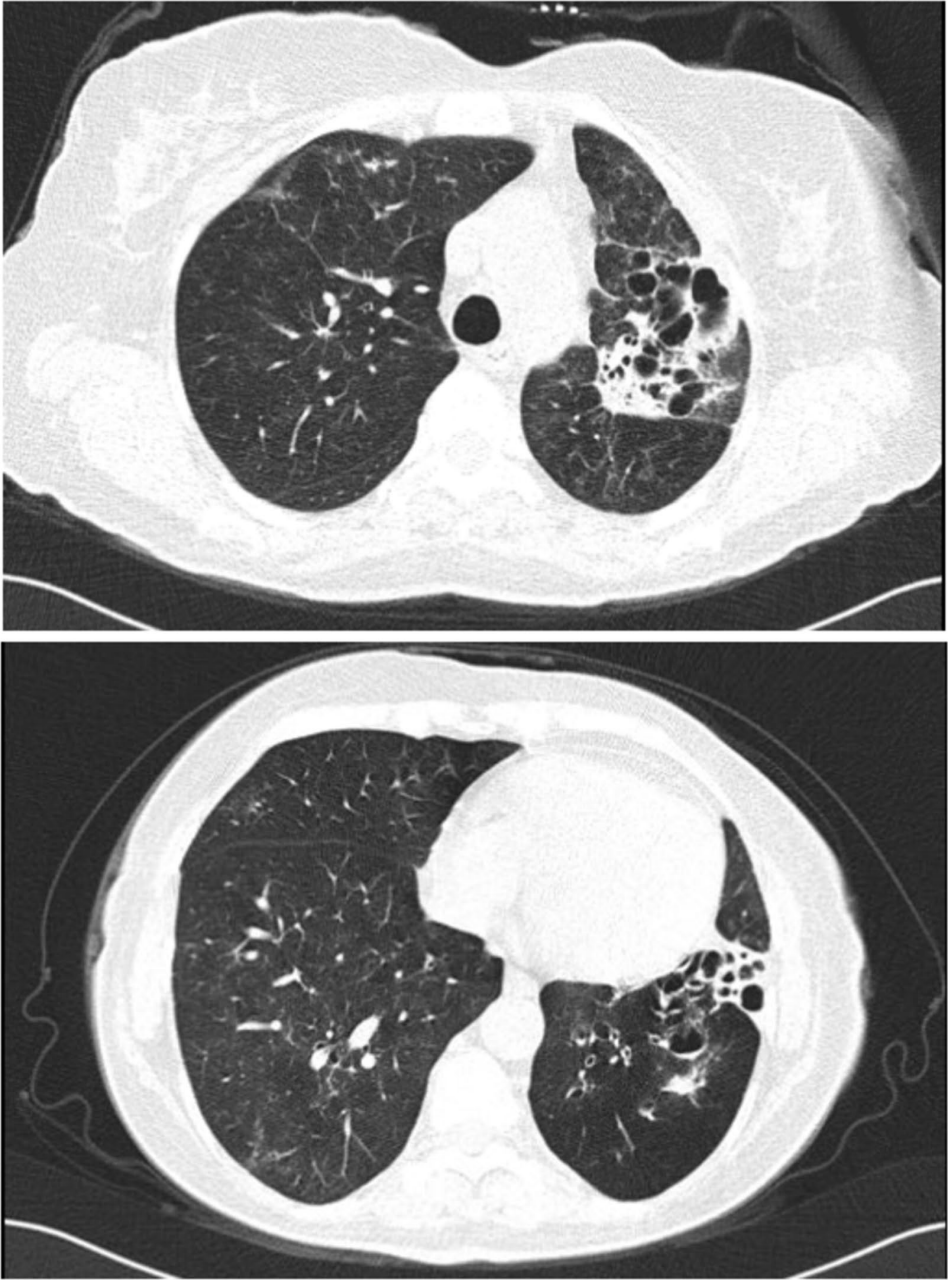
Post‐treatment HRCT showing significant resolution of lesions with remnant bronchestasis in the left lung.

The prescribed regimen was followed for a duration of 6 months, during which the patient underwent clinical and radiological evaluations showing no relapse of the condition. A post‐treatment HRCT revealed significant resolution of lesions with residual bronchiectasis in the left lung (Figure 3).

## 5 Discussion

The most prominent presentation of nocardiosis is pneumonia [7], but it can also present in different forms such as CNS or cutaneous involvement, as previously discussed. An imbalance in the immune system plays a crucial role as a predisposing factor in nocardiosis. Patients with bronchiectasis, malignancies, individuals receiving corticosteroid therapy, those with rheumatological conditions, HIV‐infected individuals, and organ transplant recipients appear to be the most commonly affected cases [4, 9]. Nocardiosis manifests as cavity formation, infiltration, nodulation, and abscesses [10]. These findings are quite similar to those observed in tuberculosis [11]. Although nocardiosis is more common in immunocompromised individuals, it is important to also consider this disease in patients with bronchiectasis and in immunocompetent patients [4, 7], as seen in our study.

In endemic regions like Iran where *M. tuberculosis* is prevalent, it is crucial to consider the possibility of another pathogen such as *Nocardia* mimicking symptoms of the disease, including fever, weight loss, and shaking chills. Both *Nocardia* and *M. tuberculosis* are acid‐fast bacteria. However, *Nocardia* is considered weakly acid‐fast and typically only shows staining on a modified Ziehl‐Neelsen stain [1, 12]. Given the resemblance in clinical and microbiological findings between *M. tuberculosis* and nocardiosis, distinguishing between the two necessitates careful attention during diagnosis. Therefore, it is essential to eliminate anchoring bias in this process. Establishing the drug sensitivity pattern of *Nocardia* species before treatment is vital. However, due to instrumental limitations and the worsening condition of the patient, treatment could not be postponed. *Nocardia asteroides* is the most prevalent species of *Nocardia* in Iran [5] and shows a high sensitivity to co‐trimoxazole, with a low resistance threshold for this antibiotic in our region [13]. Consequently, based on prior studies conducted in Iran, the patient underwent co‐trimoxazole therapy. According to the literature, the resistance pattern of *Nocardia* species in Iran includes kanamycin, cefoxitin, and isoniazid [5]. For the past 70 years, sulfonamides, particularly trimethoprim sulfamethoxazole, have been the mainstay of empiric therapy for nocardiosis [2].

This served as the basis for our empiric treatment, which yielded a favorable outcome. Additionally, as emphasized in our report, individuals with non‐responsive pneumonia often stand to benefit from bronchoscopy and specimen assessment in both immunocompetent and immunocompromised cases [14].

## Author Contributions

**Reza Ibrahimi:** conceptualization, data curation, writing – original draft. **Farid Poursadegh:** conceptualization, data curation, methodology, supervision, writing – original draft. **Mahnaz Mozdourian:** conceptualization, formal analysis. **Fariba Rezaeetalab:** data curation, investigation. **Zohre Emamdadi:** investigation, writing – original draft. **Sahar Arab Yousefabadi:** investigation, writing – original draft. **Sara Samadi:** investigation, resources, writing – review and editing.

## Ethics Statement

The study was approved by the Ethical Committee of Mashhad University of Medical Sciences. All procedures performed in this study involving human participants were in accordance with the ethical standards of the institutional and/or national research committee and with the 1964 Helsinki Declaration and its later amendments or comparable ethical standards.

## Consent

Written informed consent was obtained from the patient to publish this report in accordance with the journal's patient consent policy.

## Conflicts of Interest

The authors declare no conflicts of interest.

## Data Availability

Datasets associated with the present study are available upon reasonable request of interested researchers.
